# Caecal Bascule Volvulus: Diagnostic Challenges in a Rare cause of Large Bowel Obstruction

**DOI:** 10.1155/cris/8788042

**Published:** 2026-06-29

**Authors:** Kaotharat Balogun, Níamh M. Smyth, Charlotte Loh, Himanshu Yadav, David Beddy, Muyiwa Aremu

**Affiliations:** ^1^ Department of Surgery, Connolly Hospital, Dublin, Ireland; ^2^ Department of Surgery, Royal of College of Surgeons, Dublin, Ireland

**Keywords:** caecal bascule, caecal volvulus, large bowel obstruction, right hemicolectomy

## Abstract

Caecal bascule is the rarest form of caecal volvulus, representing 5%–20% of caecal volvulus cases and accounting for 0.2% of intestinal obstructions. Diagnosis can be delayed due to non‐specific clinical and radiological features, increasing the risk of ischaemia or perforation. Herein, we report the case of a 70‐year‐old woman who presented with vague right iliac fossa (RIF) pain, nausea and bilious vomiting. Computed tomography (CT) of the abdomen and pelvis demonstrated a mesenteric ’whirl sign’ concerning for a volvulus. Emergency laparoscopy demonstrated a distended caecum (C) folded anteriorly without torsion, consistent with caecal bascule. A right hemicolectomy with primary anastomosis was performed. The postoperative course was complicated by a small non‐drainable collection, managed conservatively with antibiotics. Histopathology showed mild vascular congestion with normal architecture and no ischaemia, malignancy or inflammatory bowel disease. This case highlights the diagnostic challenge of caecal bascule and the importance of timely surgical intervention.

## 1. Introduction

Caecal volvulus accounts for ~1%–2% of cases of large bowel obstruction [1, 2]. The bascule variant, in which the caecum (C) folds anteriorly without axial torsion, is the rarest form and represents ~5%–20% of caecal volvulus cases and only 0.2% of intestinal obstructions [1, 2]. Only a limited number of cases have been reported in the literature, reflecting its rarity. Symptoms are often non‐specific, and imaging findings may be subtle, which can delay diagnosis [3–5]. Early recognition is crucial to mitigate the risk of caecal ischaemia, perforation and clinical deterioration. Definitive management with right hemicolectomy is recommended in suitable patients to prevent recurrence and complications [3, 6, 7]. We present a case of caecal bascule in a 70‐year‐old woman, highlighting the diagnostic difficulty and successful outcome following timely surgical resection.

## 2. Case Report

A 70‐year‐old woman presented to the emergency department with a 1‐day history of gradual onset right iliac fossa (RIF) pain associated with nausea and one episode of non‐bloody bilious vomiting. She denied changes in bowel habit, with the last bowels opening within 24 h of her presentation; she was passing flatus. She denied abdominal distension, bleeding per rectum or fevers. She also denied any preceding weight loss, change in stool calibre, night sweats or other recent red‐flag symptoms.

Her past medical history is osteoporosis only. Her regular medication includes per oral calcium supplementation only. She has no known allergies. Her past surgical history was relevant for laparoscopic left oophorectomy for a teratoma 8 years prior. She is a never‐smoker, drinks fewer than 2 units a week, and has no history of current or previous recreational drug use. She lives independently at home. She had no relevant family history.

On admission, she was clinically stable, with a pulse rate of 102 beats per minute, blood pressure 96/63 mmHg, and temperature 36.0°C. Abdominal examination revealed a soft abdomen with tenderness in the RIF. There were no palpable masses and no guarding. Bowel sounds were normal. Initial biochemical and haematological investigations showed a haemoglobin of 13.8 g/dL, a white cell count of 10.7 × 10^9^/L, urea of 6.0 mmol/L, creatinine of 53 µmol/L, and a serum lactate of 1.3 mmol/L.

Contrast‐enhanced computed tomography (CT) of the abdomen and pelvis revealed a mesenteric ’whirl sign’ in the RIF, raising concern for partial mesenteric torsion, volvulus or evolving closed‐loop obstruction (Figure 1). There was no radiological evidence of obstruction or ischaemia.

**Figure 1:**
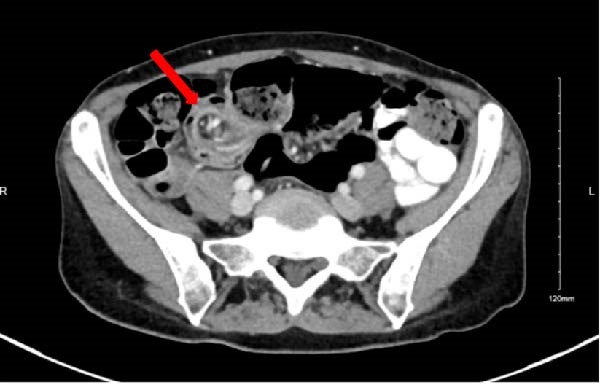
Contrast‐enhanced CT of the abdomen and pelvis demonstrating a mesenteric ’whirl sign’ in the right iliac fossa, raising suspicion for caecal volvulus.

The patient was resuscitated with intravenous fluids and was taken to the theatre for laparoscopy. Emergency diagnostic laparoscopy demonstrated the C was markedly distended and folded anteriorly over the ascending colon (AC), consistent with a caecal bascule (Figure 2). An open right hemicolectomy with a stapled primary ileocolic side‐to‐side anastomosis was performed. The patient was vitally stable throughout the operation with no immediate operative or anaesthetic complications.

**Figure 2:**
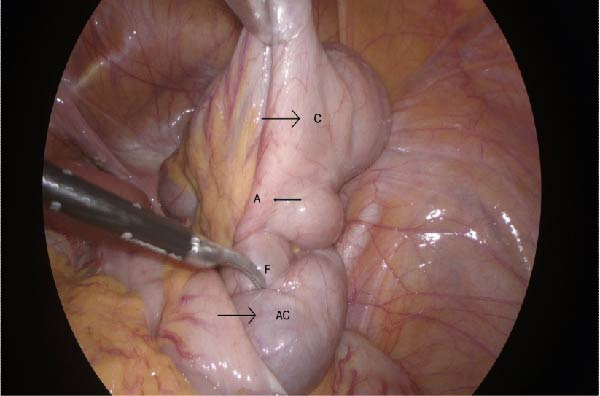
Intraoperative photograph demonstrating a markedly distended caecum (C) folded anteriorly over the ascending colon (AC) without torsion, consistent with caecal bascule. The appendix (A) is visible at the base of the caecum. *F* = anterior fold point.

She remained clinically afebrile postoperatively, despite a progressive rise in C‐reactive protein on day 4. In view of slow clinical progress and ongoing diffuse abdominal pain, a CT abdomen and pelvis demonstrated a small, non‐drainable intra‐abdominal collection in the pelvis free fluid and no signs of an anastomotic leak. She was managed conservatively with intravenous piperacillin‐tazobactam and made a complete recovery. She engaged in physiotherapy, occupational therapy and dietetic input during her hospital stay. The patient was discharged home well on post operative day 10. She was reviewed in the clinic 6 weeks afterwards and remains clinically well, with no further follow‐up required.

Histopathological analysis of the resected bowel, including terminal ileum, C, AC, A and associated mesenteric fat, demonstrated colonic mucosa with mild vascular congestion but otherwise normal architecture. The A was unremarkable, and a single benign lymph node was identified. No features of ischaemia, perforation, malignancy or inflammatory bowel disease were seen.

## 3. Discussion

Caecal volvulus accounts for ~1%–2% of large bowel obstructions and is classified into three subtypes: axial torsion (Type 1), loop‐type volvulus (Type 2), and bascule (Type 3) [3]. Bascule is the rarest subtype, representing ~5%–20% of caecal volvulus cases and only 0.2% of intestinal obstructions [1, 2]. In the caecal bascule, the C folds anterosuperiorly along a horizontal axis without mesenteric twisting, producing intermittent or partial obstruction. This mechanism may explain atypical clinical and radiological findings, including the absence of obstipation or abdominal distension and the presence of a mesenteric ’whirl sign’ despite the absence of overt obstruction or ischaemia, as demonstrated in this case [4, 5].

Diagnosis is often challenging due to non‐specific symptoms that may mimic appendicitis or gynaecological pathology, while biochemical and haematological investigations are frequently unremarkable [3]. Contrast‐enhanced CT of the abdomen and pelvis remains the diagnostic modality of choice for suspected caecal volvulus. Typical findings include caecal dilatation, abnormal caecal position and mesenteric twisting; however, these features may overlap between volvulus subtypes [4, 5]. Although the ‘whirl sign’ is classically associated with torsional volvulus, it does not reliably distinguish torsion from bascule, and definitive diagnosis often relies on operative findings [4, 5]. Whilst the whirl sign is more commonly associated with torsional volvulus, it is also a recognised CT finding in closed‐loop obstruction, reflecting mesenteric vessel twisting around a fixed point irrespective of the underlying aetiology [4, 5]. In this case, the CT report documented concern for an evolving closed‐loop obstruction in addition to volvulus; the whirl sign, therefore, most likely reflected this component rather than caecal torsion. This distinction further illustrates that a definitive diagnosis of caecal bascule relies on operative assessment rather than CT appearances alone. A high index of suspicion among surgeons and radiologists is therefore required, particularly in patients with equivocal presentations or risk factors such as caecal mobility, which may be congenital or iatrogenic following prior abdominal surgery [3].

Management must be individualised based on patient stability and intraoperative findings. Non‐resectional procedures, including tube caecostomy or caecopexy, have been described but are associated with higher recurrence rates. Resection with right hemicolectomy provides definitive management and is generally recommended in clinically stable patients with viable bowel, as it minimises recurrence and addresses potential underlying pathology [3, 6–8]. In this case, timely surgical intervention allowed definitive treatment prior to the development of bowel compromise, resulting in a favourable outcome. Our patient underwent right hemicolectomy, and histology confirmed the absence of ischaemia or perforation, highlighting the importance of timely surgical intervention to prevent bowel compromise. The postoperative course was notable for only a minor complication, managed conservatively, and she was discharged home on day 10 with good functional recovery.

This case illustrates several important clinical features. Firstly, caecal bascule can present without classical features of large bowel obstruction and in patients with no known risk factors, which requires surgeons and radiologists to maintain a broad differential when evaluating RIF pain. Secondly, prompt CT imaging is crucial for diagnosis. Lastly, surgical resection remains the most effective treatment, preventing recurrence, managing a potential underlying pathology and avoiding life‐threatening complications.

## 4. Conclusion

Caecal bascule is a rare but important differential diagnosis for acute RIF pain. Diagnosis may be difficult due to non‐specific clinical presentation and overlapping imaging features. CT imaging can provide valuable diagnostic clues, but operative assessment remains definitive. Early recognition and timely surgical resection are essential to prevent progression to ischaemia, perforation and recurrence.

## Funding

This research did not receive any specific grant from funding agencies in the public, commercial or not‐for‐profit sectors.

## Ethics Statement

The authors assert that all procedures contributing to this work comply with the Helsinki Declaration of 1975, as revised in 2008.

## Consent

The participant consented to the submission of the case report to the journal.

## Conflicts of Interest

The authors declare no conflicts of interest.

## Data Availability

The data that support the findings of this study are available upon request from the corresponding author. The data are not publicly available due to privacy or ethical restrictions.
